# Sistematización de una intervención cognitivo – comunicativa basada en reminiscencia para adultos mayores

**DOI:** 10.1590/2317-1782/20232022152pt

**Published:** 2023-12-04

**Authors:** Pedro García Montenegro, Elenir Fedosse, Gabriel Urrutia Urrutia

**Affiliations:** 1 Departamento de Ciencias de la Fonoaudiología, Facultad de Ciencias de la Salud, Universidad de Talca – UTALCA – Talca (VII Región del Maule), Chile.; 2 Programa de Pós-graduação em Distúrbios da Comunicação Humana, Universidade Federal de Santa Maria – UFSM – Santa Maria (RS), Brasil.; 3 Departamento de Ciencias de la Fonoaudiología, Facultad de Ciencias de la Salud, Universidad de Talca – UTALCA – Talca (VII Región del Maule), Chile.

**Keywords:** Memoria Autobiográfica, Terapia de Reminiscencia, Fonoaudiología, Bienestar, Eficacia Cognitiva, Envejecimiento

## Abstract

**Objetivo:**

Determinar el efecto del programa de intervención cognitivo - comunicativo basado en la reminiscencia (PECC-R) sobre el procesamiento cognitivo global y la autopercepción de bienestar subjetivo.

**Método:**

En una muestra intencional de 100 adultos mayores autovalentes, 65 conformaron el grupo estudio y 35 el grupo control. El programa se administró en el grupo de estudio. Mediante la prueba de Wilcoxon, se compararon las medidas de resultado de eficacia cognitiva global (MMSE) y bienestar subjetivo (SWLS) antes y después del programa, Asimismo, se compararon las diferencias del pre y postest de ambos grupos mediante U de Mann Whitney.

**Resultados:**

Se observó una diferencia estadísticamente significativa entre el pre y post test del SWLS en el grupo estudio, no así en el grupo control. Este resultado se replicó en la variable eficacia cognitiva global. Se observó una diferencia entre los grupos en ambas medidas, con mayor beneficio en el grupo que recibió la intervención.

**Conclusión:**

El PECC-R constituye una alternativa viable para la estimulación cognitivo-comunicativa de orden funcional. La evidencia de las diferencias en las actuaciones respalda su utilidad y validez para la intervención en Atención Primaria u otros contextos similares

## INTRODUCCIÓN

Existe una gran heterogeneidad para las alternativas de estimulación cognitiva orientadas hacia la población de adultos mayores, todas ellas centradas en incidir positivamente sobre los factores de riesgo para el deterioro cognitivo y la demencia^(1)^. Estas condiciones clínicas, que en conjunto provocan pérdida de autonomía y funcionalidad, son altamente prevalentes en este grupo de edad^(2)^ y de interés prioritario para la investigación internacional en diversas áreas del conocimiento^(3)^.

En general, las estrategias de estimulación cognitiva suelen focalizarse en los déficits de memoria involucrados en la vejez, particularmente en su aspecto más episódico , utilizando a la vez diferentes medidas de objetivación para ello^(4)^.

Las intervenciones basadas en reminiscencia, revisión de vida o recuerdos autobiográficos son actividades que presentan algunas particularidades:

Hasta ahora, tienen múltiples fuentes de probada eficacia en poblaciones clínicas, lo que ha permitido incrementar estudios de revisiones sistemática y metaanálisis en diversos de sus beneficiarios, pero como los mismos revisores reportan, con escasa homogeneidad en sus procedimientos^(5-11)^. A continuación enumeramos algunas de las características de este tipo de investigación:

1. Como parte de las intervenciones de carácter psicosocial, estos trabajos han privilegiado el uso de medidas de resultados (*outcomes*) por dominio o función, situación que se condice con la evolución histórica que ha experimentado esta línea de trabajo desde uno de sus pioneros^(12)^. En concreto, los programas justifican diferentes actividades y estrategias de trabajo pero también escasamente avanzan desde las medidas instrumentales hacia procesos o características comportamentales de mayor validez funcional, tales como la interacción social y comunicativa o de autopercepciones tales como medidas de felicidad, calidad de vida o bienestar subjetivo que favorecen estrategias positivas de afrontamiento durante la vejez^(13)^. Esta situación constituye un enorme desafío, dadas las complejidades inherentes y la relevancia que suponen dichos indicadores;
2. Aunque los datos proporcionados no siempre son del todo concluyentes la replicabilidad de los programas no siempre es posible dado que los protocolos de procedimiento y los contenidos suelen informarse de modo genérico y rara vez se encuentran totalmente disponibles. Asimismo;
3. Se reconoce la necesidad de avanzar por la vía de estudios cualitativos por la riqueza intrínseca de los métodos, su flexibilidad y la accesibilidad diferenciada hacia grupos de interés particular.

El presente trabajo persigue promover una estimulación cognitiva integral, que sea aplicable en diferentes espacios de participación comunitaria, de bajo costo operacional y que además contribuya a favorecer aspectos positivos de la autoimagen de las personas envejecidas, por lo que se decidió elaborar un Programa de Estimulación Cognitivo - Comunicativa basado en Reminiscencias - (PECC-R).

## METODOLOGÍA

La formulación y desarrollo del PECC-R estableció como punto de partida el análisis de la oferta programática en estimulación cognitivo-comunicativa para adultos mayores que son beneficiarios de los programas de atención gubernamentales, ejecutados por unidades de Atención Primaria de Salud u Organizaciones Comunitarias.

Concordantemente, el PECC-R, fue desarrollado considerando un diálogo que involucró a los diferentes interesados, relevando como prioritario la búsqueda de una estrategia de carácter interactivo (dimensión comunicativa), que permitiera trabajar sobre funciones cognitivas específicas (memoria episódica autobiográfica), pero que además aportará con un sentido de identificación y continuidad temporal entre los hechos del pasado, la situación actual y la prospección de futuro. Esto orientó respecto a un modelo de reminiscencia de carácter integrativo^(14,15)^ que además se viera favorecido del trabajo colaborativo entre los facilitadores y los participantes^(16-18)^.

Así, el PECC-R fue revisado y consensuado por un panel de 12 profesionales fonoaudiólogos vinculados con el ámbito de intervención, quienes además poseían al menos cinco años de experiencia clínica en la atención de adultos mayores. Dicho panel valoró, mediante un cuestionario con un escalamiento tipo Likert, la estructura general de trabajo, la metodología utilizada, la organización interna y de progresión de las sesiones del programa y su utilidad para la estimulación cognitivo – comunicativa funcional basada en reminiscencias^(19)^.

**Figura 1:**
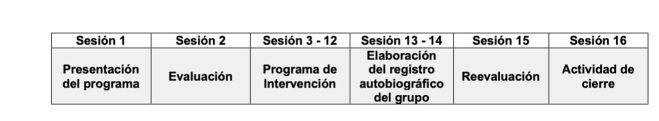
Estructura temporal y de progresión del PECC-R

Incorporada la información de los jueces, la estructura definitiva del PECC-R consta de un total de 16 sesiones, con periodicidad de una reunión semanal y una duración de aproximadamente una hora. En la primera sesión, se realizan las presentaciones entre facilitadores y participantes. Además, se busca resolver cualquier duda que exista respecto a las actividades que se desarrollarán. En la sesión 2 y la sesión 15 se realizan las evaluaciones pre y post respectivamente. Las sesiones 3 a 12 (10 en total), están destinadas a recuperar y profundizar en los relatos de las reminiscencias, las que son facilitadas mediante actividades específicas. En las sesiones 13 y 14 se elabora un producto consensuado como grupo (registro biográfico). Finalmente, la sesión 16 se destina al cierre del programa, con actividades lúdicas y premiaciones. La Figura 1 presenta un esquema temporal de la estructura del programa.

Cada actividad está a cargo de un facilitador principal debidamente entrenado, quien puede ser asistido por un máximo de otros dos facilitadores, pero de menor responsabilidad en la conducción de la actividad. El grupo de trabajo no debe exceder los 12 beneficiarios, para mantener siempre el control de la situación y el manejo de imprevistos.

**Figura 2:**
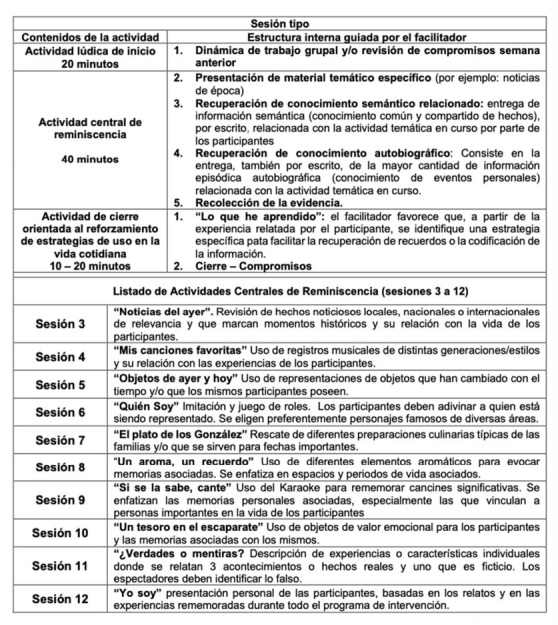
Organización del PECC-R, estructura interna de una sesión tipo y temáticas de reminiscencia por sesión

Las sesiones de intervención (sesiones 3 a 12 en la Figura 1), permiten una progresión en la profundidad de los recuerdos. De este modo, las cuatro primeras sesiones (3 a 6), tienen temáticas de orden general, tales como: hechos o eventos de tipo histórico-social de la comunidad local – nacional y/o internacional. Las cuatro sesiones siguientes (7 a 10), van avanzando progresivamente hacia recuerdos más personales. Es preciso señalar que todas estas sesiones poseen la misma organización interna, variando solo la actividad lúdica inicial y el tipo contenido de la actividad de reminiscencia, tal como se grafica en la Figura 2. Finalmente, las sesiones 11 y 12, se enfocan directamente en la identificación de eventos relevantes individuales.

Cabe destacar que, aunque las actividades se orientan hacia la recuperación de eventos o vivencias de valencia positiva y constructiva para el individuo, algunos de ellos pueden evocar situaciones neutras o negativas. En este sentido es fundamental el rol del facilitador, quien debe poseer las competencias necesarias para contener, apoyar y manejar la situación, dentro del grupo e individualmente. Se considera un protocolo de seguimiento para estos casos, instruyendo específicamente en el uso del teléfono ante cualquier consulta o duda que se tenga respecto a los sentimientos o emociones que pudiese experimentar después de alguna de las sesiones de intervención. El participante es informado al momento del consentimiento de esto, así como de dos llamadas de seguimiento que se realizan a las 24 y 48 horas de las sesiones 3 y 4 respectivamente.

Durante el contacto telefónico se aplica un breve cuestionario sobre el estado general del participante. Los resultados de las preguntas orientan dos decisiones: visita al centro para derivación especializada o seguimiento de control por parte del programa comunitario. Se insiste siempre en que cualquier duda o consulta, la persona puede acercarse en horarios de oficina al centro clínico o contactarse telefónicamente para solicitar una cita.

Las sesiones 13 y 14 del PECC-R se destinan a generar un registro individual y/o grupal de la actividad, eligiendo los participantes si trabajan con biografías individuales y/o narrativas relacionadas con la colectividad. Estos registros pueden involucrar elementos de baja tecnología, como lo son los libros de memorias hasta elementos multimedia, donde los protagonistas relevan aquellos aspectos de las memorias individuales o colectivas que han sido recuperadas. En tal sentido, los facilitadores apoyan la elaboración del material, el que es generalmente presentado en la sesión de cierre, momento donde se ha extendido la invitación a la participación a familiares o amistades de los participantes y/o a diferentes actores sociales de la comunidad.

### Muestreo

La selección de los participantes para esta investigación se basó en un procedimiento de muestreo dirigido, por conveniencia. En primer lugar, se asistió a cinco centros que demostraron interés y sensibilización ante la actividad propuesta. En ellos se explicó la naturaleza del programa, temporalidad, número de sesiones y actividades que se realizarían. Posteriormente a esto se enlistó a los interesados, con los que se implementó un protocolo de entrevista que consideró las siguientes etapas: i) anamnesis y toma de datos personales; aplicación de pruebas de exclusión – condición de entrada y iii) firma de consentimiento informado.

### Criterios de Inclusión

- Tener una edad mayor o igual a 60 años;
- Contar con al menos ocho años de escolaridad (ya sea continua o discontinua);
- Estar inscrito en la unidad de atención primaria de salud de su comuna;
- Participar activamente (por lo menos con un año de antigüedad) en alguna agrupación social comunitaria;
- Encontrarse retirado de sus actividades laborales (jubilados);
- Poseer una acuidad auditiva y visual acorde a las actividades solicitadas o en su defecto, contar con las adaptaciones correspondientes.
  ● Acreditar un estatus cognitivo, funcional y emocional dentro de valores típicos:
  - Puntaje 1 en la Global Deterioration Scale – GDS^(20)^;
  - Puntuaciones mayores o iguales a 23 puntos en el Minimental State Examination - MMSE^(21)^;
  - Puntuaciones menores o iguales a seis en el Pfeffer Functional Activities Questionnaire - PFAQ^(21)^;
  - Puntuaciones menores o iguales a cuatro en el Cuestionario de Salud General de Goldberg-12^(22)^;
  - Sin antecedentes médicos de: accidente cerebrovascular, traumatismo encéfalo-craneano, enfermedades neurológicas progresivas – neurodegenerativas y/o trastornos neuropsiquiátricos;
  - Sin encontrarse en tratamiento con psicofármacos;
    o Comprometer una participación (asistencia) no menor a un 80% de las sesiones de aplicación del programa propiamente tal (esto corresponde a 8 sesiones. No se consideran para este cálculo las sesiones de evaluación pre y post intervención).

Atendiendo a los criterios de selección, el grupo de estudio incluyó un total de 65 adultos mayores típicos, de ambos sexos. Se descartó las participación de aquellos que reportaron: 1) alguna enfermedad psiquiátrica diagnosticada por médico en los últimos seis meses (a quienes se les recomendó seguir su tratamiento y participación en otras actividades de estimulación en el centro al que asistían), y 2) personas con limitaciones de comunicación severas, a quienes se les ofreció la posibilidad de actividades personalizadas en el centro de clínicas propio de la Universidad de Talca, en la misma ciudad.

Por su parte, el grupo control estuvo compuesto por un total de 35 adultos mayores típicos de ambos sexos y de la misma ciudad, inscritos en otras agrupaciones comunitarias y que participaban de actividades de esparcimiento y acondicionamiento físico u otros programas que no estuviesen basados en reminiscencia. La Tabla 1 resume los antecedentes sociodemográficos y clínicos de ambos grupos.

**Tabla 1:** Antecedentes sociodemográficos y clínicos de los participantes adultos mayores

Parámetros	Grupo estudio (n= 65)	Grupo control (n=35)	P value*
**Edad (años), Media ± DE**	73,92 ± 8,01	74,09 ± 7,96	***p> 0,05***
**Escolaridad (años), Media ± DE**	7,37 ± 2,80	8,23 ± 2,65	***p> 0,05***
**Género, frecuencia absoluta (%)**	M= 14 (21,54%)	M= 6 (17,14%)	--
	F= 51 (78,46%)	F= 29 (82,86%)	
Percepción de nivel socioeconómico	B= 45 (69,23%)	B= 25 (71,43%)	--
	M= 20 (30,77%)	M= 10 (28,57%)	
Funcionalidad en AVD			
**PFAQ, Media ± DE**	2,71 ± 1,02	2,15 ± 0,86	***p> 0,05***
Funcionamiento cognitivo global			
**MMSE, Media ± DE**	26,72 ± 2,05	27,17 ± 1,72	***p> 0,05***

* Valores de significancia estadística para la prueba U-Mann Whitney; (N.S) = p> 0,05, diferencias No Significativas

**Subtítulo:** DE= Desviación estándar; B= nivel socioeconómico bajo; M= nivel socioeconómico medio; MMSE= Mini Mental State Examination; M= másculino; F= femenino; AVD= Actividades de la Vida Diaria; PFAQ= versión Chilena del Pfeffer Functional Activities Questionnaire

### Consideraciones éticas

El PECC-R” se aplicó después de la aprobación otorgada por el Comité de Ética en Pesquisa de la Universidade federal de Santa Maria, Brasil, Folio Nº 3.000.149. Todos los participantes firmaron además un formulario de consentimiento libre e informado.

### Análisis estadístico

Se establecieron dos medidas de resultado: bienestar subjetivo percibido, y eficacia cognitiva global. La primera constituye una medida de autopercepción mientras que la segunda es el resultado de una prueba de tamizaje cognitivo. Todos los datos se analizaron con el Paquete Estadístico para las Ciencias Sociales SPSS, en su versión 25, para Apple.

1. **Bienestar subjetivo**: para establecer si hubo o no diferencias en la autopercepción del bienestar subjetivo luego de implementar la intervención, se consideró el puntaje obtenido en la Escala de Satisfacción con la Vida - SWLS^(23)^. La SWLS incluye cinco preguntas y un escalamiento Likert de siete, por lo que el puntaje mínimo es de cinco puntos y el máximo de 45. No posee punto de corte. Se asumen valores altos con bienestar subjetivo alto. Al igual que la medida anteriormente descrita, se realizó una comparación intra-grupal con el estadígrafo de Wilcoxon y una comparación intergrupal con el estadígrafo U de Mann Whitney.
2. **Eficacia Cognitivo Global**: se consideró el resultado obtenido en la versión Chilena del MMSE en asociación con el PFAQ^(21)^ cuyo punto de corte para diferenciar personas con deterioro cognitivo es de 21/22 puntos. Para observar las ganancias observadas en el funcionamiento cognitivo global, se comparó el resultado del MMSE antes de implementar el programa de intervención basado en reminiscencia y luego de este. Además, para establecer si las ganancias observadas se atribuían a dicho programa, se comparó las diferencias observadas entre el postest y el pretest del grupo estudio y grupo control mediante la prueba U de Mann Whitney.

## RESULTADOS

### Proceso de validación del PECC-R

Los jueces concordaron que tanto las sesiones de trabajo propuestas como la estructura general del programa permiten contribuir a la estimulación cognitivo – comunicativa de personas mayores, en el marco de un modelo funcional (W=0,762; p=0,019).

### Evidencia de eficacia del PECC-R

#### Análisis de las diferencias entre el pretest y postest en la autopercepción de bienestar subjetivo

En el resultado pretest del SWLS, el grupo estudio (n=65) obtuvo un rango de puntuaciones que variaron entre 10 y 35 puntos, con una puntuación media de 22,82 puntos y una desviación estándar de 6,14 puntos. Asimismo, este grupo alcanzó un rango entre 17 y 35 puntos, con una media de 25,82 puntos y una desviación estándar de 4,38 puntos en el postest. La prueba de Wilcoxon da cuenta de que dicha diferencia fue estadísticamente significativa (W= -6,33; p= 0,000).

En el pretest del SWLS del grupo control (n= 35), el puntaje mínimo fue de 12 y el máximo de 35 puntos, con una media de 23,74 puntos y una desviación estándar de 6,71. En el postest, el rango de puntuaciones osciló entre 12 y 34, con una puntuación media de 23,74 puntos y una desviación estándar de 6,71. Al analizar comparativamente los resultados, la prueba de Wilcoxon muestra que no hay una diferencia estadísticamente significativa entre ambas puntuaciones (W= -0,37; p= 0,708).

Tanto la información del grupo estudio como la del grupo control respecto al bienestar subjetivo, se resume en la Tabla 2.

**Tabla 2:** Comparación de la percepción de bienestar subjetivo antes y luego de la intervención basada en reminiscencia entre ambos grupos de participantes

	**Medida de resultado SWLS**	**Pretest**	**Postest**	P value*
Grupo estudio (n= 65)	rango	10 – 35	17 – 35	p= 0,000**
	media ± DE	22,82 ± 6,14	25,82 ± 4,38	
Grupo control (n= 35)	rango	12 – 35	12 – 34	*p> 0,05.*
	media ± DE	23,74 ± 6,71	23,6 ± 4,79	

* Valores de significancia para la prueba Wilcoxon; (N.S)= p> 0,05, diferencias No Significativas;

** p< 0,05: diferencias significativas

**Subtítulo:** DE= Desviación estándar; SWLS= Escala de Satisfacción con la Vida

#### Análisis de las diferencias entre el pretest y postest en el funcionamiento cognitivo global

El grupo estudio (n= 65) obtuvo un rango de puntuaciones que variaron entre 24 y 30 puntos, con una puntuación media de 26,72 puntos y una desviación estándar de 1,68 en el resultado pretest del MMSE. Asimismo, en el postest alcanzó un rango entre 24 y 30 puntos, con una media de 27,20 puntos y una desviación estándar de 1,87. La prueba de Wilcoxon mostró que dicha diferencia fue estadísticamente significativa (W= -4,43; p= 0,000).

Por otro lado, en el grupo control (n= 35), el puntaje mínimo fue de 25 y el máximo de 30 puntos, con una puntuación media de 27,17 puntos y una desviación estándar de 1,72 en el resultado pretest del MMSE. Del mismo modo, en el postest el rango de puntuaciones osciló entre 24 y 30, con una media de 27,29 puntos y una desviación estándar de 2,34. Al analizar comparativamente dichos resultados, la prueba de Wilcoxon muestra que entre ambas puntuaciones no hay una diferencia estadísticamente significativa (W= -1,21; p= 0,225).

Tanto la información del grupo estudio como la del grupo control respecto a la eficacia cognitiva global, se resumen en la Tabla 3.

**Tabla 3:** Comparación del procesamiento cognitivo global antes y luego de la intervención basada en reminiscencia entre ambos grupos de participantes

	**Medida de resultado MMSE**	**Pretest**	**Postest**	P value*
Grupo estudio (n= 65)	rango	24 – 30	24 – 30	p< 0,05**
	media ± DE	26,72 ± 2,05	27,20 ± 1,87	
Grupo control (n= 35)	rango	25 – 30	24 – 30	*p> 0,05*
	media ± DE	27,17 ± 1,72	27,29 ± 2,34	

* Valores de significancia para la prueba Wilcoxon; (N.S)= p> 0,05, diferencias No Significativas;

** p< 0,05: diferencias significativas

**Subtítulo:** DE= Desviación estándar; MMSE= MiniMental State Examination

Finalmente, al comparar las diferencias observadas en los resultados del pretest y postest del MMSE entre el grupo estudio y control, la prueba U de Mann Whitney muestra que existen diferencias estadísticamente significativas entre ambos grupos (U= 587; p= 0,002).

## DISCUSIÓN

Aunque desde una lectura muy superficial el diseño y la formulación de actividades relacionadas con eventos autobiográficos solo esté centrado en procurar la facilitación de estos, dichas intervenciones poseen diferentes dimensiones y niveles de complejidad, inherentes a su estructura y organización, situación que junto a la heterogeneidad propia de los participantes y los objetivos que son perseguidos, plantean importantes desafíos a fin de sostener la rigurosidad metodológica^(24)^.

Puesto que una de las limitaciones más discutidas con respecto a estos programas la constituye esta variabilidad intrínseca^(5)^, es fundamental destacar la importancia de la protocolización y sistematicidad en la aplicación de todos los elementos relacionados con la estructura de las sesiones de intervención. Asimismo, debe revelarse que en este tipo de actividades siempre han de ser considerados aspectos de orden ético, tanto para el manejo y desarrollo de los actos de reminiscencia, como para orientar posibles soluciones a implementar ante cualquier eventualidad^(25)^.

Es indudable que las medidas de eficacia cognitiva global y bienestar subjetivo obtenidas constituyen una referencia de orden y complejidad basal, considerando que se trata de evaluaciones de tamizaje y de constructos unidimensionales. Se requiere, por una parte, avanzar en estrategias de evaluación de mayor amplitud y sofisticación, sin desconocer la necesidad de implementar registros sistemáticos de medidas relativas al autocuidado, la calidad de vida y el bienestar subjetivo, tal como se aplica en poblaciones clínicas^(26)^.

Aunque una limitación de esta investigación se encuentra en la ausencia de indicadores de cambio en las conductas lingüísticas, es posible dar cuenta de diferencias cuantificables en la complejidad sintáctica de los discursos y en la cantidad de unidades de contenido, así́ como una mayor especificidad en la construcción de los recuerdos. Dichos antecedentes hasta la fecha se han establecido mediante la comparación de unidades discursivas intersujetos.

De todos modos, es importante destacar que desde los modelos clásicos de competencia comunicativa - discursiva^(27)^ y las concepciones actuales de participación en el diseño de programas e intervención^(28,29)^, la relevancia de las narrativas personales pueden visualizarse como alternativas altamente funcionales para favorecer la identidad, el empoderamiento y la mantención de la funcionalidad en los adultos mayores, ampliando los alcances desde las poblaciones clínicas hacia el desarrollo de actividades de prevención y promoción en la neurodiversidad, con claros lineamientos hacía reconocer en la interacción comunicativa un derecho inalienable en la adultez mayor^(30)^, así como en cualquier etapa del ciclo vital.

## CONCLUSIÓN

El PECC-R es una alternativa viable de estimulación cognitivo – comunicativa de orden funcional que requiere una implementación sólida y bien cuidada, considerando para los beneficiarios como requisito básico el contar con habilidades de interacción, con independencia de su nivel educacional; asimismo, el contar con evidencia de incrementos estadísticamente significativos en los desempeños de los participantes del programa, avalan su utilidad y eficacia para la intervención. Consecuentemente, este programa es posible de aplicar en Atención Primaria de Salud, así como en otros contextos asistenciales similares.
