# Supplementary material for: Sistematización de una intervención cognitivo – comunicativa basada en reminiscencia para adultos mayores
Source: Codas. 2023 Dec 4;35(6):e20220152. [Article in Spanish] doi: 10.1590/2317-1782/20232022152pt (PMC10903332; doi:10.1590/2317-1782/20232022152pt)
Supplement: Translation

# Translation: Systematization of a cognitive-communicative intervention based on reminiscence for older adults

**DOI:** 10.1590/2317-1782/20232022152en

**Authors:** Pedro García Montenegro, Elenir Fedosse, Gabriel Urrutia Urrutia

**Affiliations:** 1 Departamento de Ciencias de la Fonoaudiología, Facultad de Ciencias de la Salud, Universidad de Talca – UTALCA – Talca (VII Región del Maule), Chile.; 2 Programa de Pós-graduação em Distúrbios da Comunicação Humana, Universidade Federal de Santa Maria – UFSM – Santa Maria (RS), Brasil.; 3 Departamento de Ciencias de la Fonoaudiología, Facultad de Ciencias de la Salud, Universidad de Talca – UTALCA – Talca (VII Región del Maule), Chile.

**Keywords:** Autobiographical Memory, Reminiscence Therapy, Speech and Language Therapy, Well-Being, Cognitive Efficacy, Aging

## Abstract

**Purpose:**

To determine the effect of the reminiscence-based cognitive-communicative intervention program (CCSP-R) on global cognitive processing and self-perception of subjective well-being.

**Methods:**

A purposive sample of 100 self-care elderly adults, with 65 composing the study group and 35 in the control group. The study group was subjected to the proposed program. The Wilcoxon test compared the outcome measures of global cognitive efficacy (MMSE) and subjective well-being (SWLS) before and after the program, whereas the Mann-Whitney U test compared the pre- and post-test differences between the two groups.

**Results:**

A statistically significant difference was found between the pre- and post-test of the SWLS in the study group, but not in the control group. This result was replicated in the global cognitive efficacy variable. A difference between the groups occurred in both measures, with greater benefit for the group that received the intervention.

**Conclusion:**

The CCSP-R is a viable alternative for cognitive-communicative stimulation of functional order. The evidence of differences in performance supports its usefulness and validity for intervention in primary care or other similar contexts.

## INTRODUCTION

There is great heterogeneity in the cognitive stimulation alternatives aimed at the elderly population, all of them focus on positively influencing risk factors for cognitive impairment and dementia^(1)^. These clinical conditions, which together cause loss of autonomy and functionality, are highly prevalent in this age group^(2)^, representing a primary concern for international research in various areas of knowledge^(3)^.

In general, cognitive stimulation strategies tend to focus on memory deficits involved in old age, particularly in its more episodic aspect, simultaneously using different objectification measures^(4)^.

Reminiscence-based interventions, life reviews, or autobiographical memories have some particularities. Until now, they present multiple sources of proven efficacy in clinical populations, which has allowed increasing studies of systematic reviews and meta-analyses in various of their beneficiaries; however, as the reviewers themselves report, there is little homogeneity in their procedures^(5-11)^. Below we list some of the features of this type of research:

1. As part of psychosocial interventions, these works have favored the use of outcome measures by domain or function, which is consistent with the historical evolution of this research line since its pioneers’ work^(12)^. Specifically, although they justify different activities and work strategies, these programs have also hardly advanced from instrumental measures towards processes or behavioral characteristics of greater functional validity, such as social and communicative interaction or self-perceptions, such as measures of happiness, quality of life, or subjective well-being that favor positive coping strategies for older adults^(13)^. Such a scenario is a huge challenge given the inherent complexities and relevance of these indicators;
2. Although the data provided are not always completely conclusive, these programs cannot always be replicated since their procedural protocols and contents are usually reported generically and are rarely fully available;
3. In addition, there is a clear need to advance along the path of qualitative studies due to the intrinsic richness of the methods, their flexibility, and the differentiated accessibility towards groups of particular interest.

This paper seeks to promote a comprehensive cognitive stimulation, which is applicable in different spaces of community participation at a low operational cost, in addition to contributing to favoring positive aspects of the self-image of the older population. For such a purpose, we chose to develop a Reminiscence-Based Program for Cognitive-Communicative Stimulation – (CCSP-R).

## METHODOLOGY

The CCSP-R was formulated and developed as a starting point to analyze the program proposal for cognitive-communicative stimulation in older adults benefited by government care programs provided by Primary Health Care units or Community Organizations.

Accordingly, the CCSP-R was developed considering a dialogue that involved the different stakeholders focusing on searching for an interactive strategy (communicative dimension), which would allow working on specific cognitive functions (autobiographical episodic memory). Furthermore, the instrument will contribute with a sense of identification and temporal continuity among the events of the past, the current situation, and the prospect of the future. This led to an integrative reminiscence model^(14,15)^ that would also benefit from collaborative work between facilitators and participants^(16-18)^.

Thus, the CCSP-R was reviewed and accepted by a panel of 12 speech-language therapists associated with the intervention field , who also had at least five years of clinical experience in the care of older adults. This panel used a questionnaire with a Likert-type scaling to assess the general work structure, methodology, internal organization, and progression of the program sessions, in addition to its usefulness for reminiscence-based functional cognitive-communicative stimulation^(19)^.

Incorporating the information by the examiners, the final structure of the CCSP-R consists of a total of 16 sessions, with a weekly meeting and a duration of approximately one hour. The first session involves presentations between the facilitators and participants and focused on resolving any potential doubts regarding the subsequent activities. Sessions 2 15 cover the pre- and post-assessments, respectively. Sessions 3 to 12 (10 in total) are designed to recover and deepen the stories of reminiscences through specific activities. In sessions 13 and 14, a product is elaborated as a group (biographical record). Finally, session 16 completes the program with recreational activities and awards. Figure 1 shows a time frame of the program structure.

**Figure 1:** Temporal structure and progression of the CCSP-R

A properly trained main facilitator is responsible for each activity , who can be assisted by up to two other facilitators, who, in turn, do not have the same responsibility in conducting the activity. The working group should not exceed 12 beneficiaries to maintain control of the situation and manage unforeseen events.

The intervention sessions (sessions 3 to 12 in Figure 1) allow progressing toward the depth of memories. Thus, the first four sessions (3 to 6) have themes of a general order, such as facts or events of a historical-social nature in the local community – national and/or international. The next four sessions (7 to 10) advance progressively toward more personal memories. It is worth noting that all these sessions have the same internal organization, varying only the initial playful activity and the type of the reminiscence activity, as shown in Figure 2. Finally, sessions 11 and 12 focus directly on identifying individual relevant events.

**Figure 2:** Organization of the CCSP-R internal structure of a typical session and reminiscence themes per session

Although the activities are oriented towards recovering positive and constructive events or experiences for the individual, some of them can evoke neutral or negative situations. Thereby, the facilitator has a fundamental role and must hold the necessary skills to contain, support, and manage the situation, both within the group and individually. In these cases, a follow-up protocol is considered specifically instructing on the use of the telephone in the event of any query or doubt regarding the feelings or emotions that may be experienced after any of the intervention sessions. The participant is informed at the time of their consent and two follow-up calls are made 24 and 48 hours after sessions 3 and 4, respectively.

Upon telephone contact, a brief questionnaire is applied on the general condition of the participant. The results of the questions help decide between a visit to the center for specialized referral and a follow-up control by the community program. The individual is reinforced that they can approach the clinical center during office hours or contact by phone to request an appointment for any doubt or query.

Sessions 13 and 14 of the CCSP-R are intended to generate an individual and/or group record of the activity, choosing the participants based on their work with individual biographies and/or narratives related to the community. These records may involve low-tech elements, from memory books to multimedia elements, where the protagonists reveal those aspects of individual or collective memories that have been recovered. In this sense, the facilitators support the preparation of the material, which is generally presented in the closing session, when the invitation to participate has been extended to family members or friends of the participants and/or to different social actors in the community.

### Sample

The participants were selected based on a directed sampling procedure by convenience. First, five centers that showed interest and awareness of the proposed activity were assisted. They were instructed on the nature of the program, timing, number of sessions, and activities to be performed. Subsequently, the interested parties were enlisted by implementing an interview protocol that considered the following stages: i) anamnesis and personal data collection; application of exclusion tests – entry condition, and iii) signing of the informed consent form.

### Inclusion criteria

- **To be 60 years or older.**
- To have at least eight years of education level (either continuous or discontinuous);
- To be enrolled in the primary health care unit of their community;
- To participate actively (for at least one year) in a community social group;
- To be retired from their work activities (retired);
- To have the auditory and visual acuity required for the requested activities or, otherwise, the corresponding adaptations;
  ● To demonstrate a cognitive, functional, and emotional status within typical values, as follows:
  - Score 1 on the Global Deterioration Scale – GDS^(20)^;
  - Scores greater than or equal to 23 points on the Mini Mental State Examination – MMSE^(21)^;
  - Scores less than or equal to six on the Pfeffer Functional Activities Questionnaire – PFAQ^(21)^;
  - Scores less than or equal to four on the Goldberg General Health Questionnaire-12^(22)^;
  - No medical history of cerebrovascular accident, brain injury, progressive neurological diseases – neurodegenerative and/or neuropsychiatric disorders;
  - Not being in treatment with psychotropic drugs;
    o To participate (attendance) in at least 80% of the application sessions of the program itself (corresponding to 8 sessions). Pre- and post-intervention assessment sessions are not considered for this calculation.

Based on the selection criteria, the study group included a total of 65 typical older adults of both genders. Participants who reported the following conditions were disregarded: 1) some psychiatric illness diagnosed by a doctor in the last six months (with recommendation to continue their treatment and participate in other stimulation activities at the center they attended), and 2) people with severe communication limitations (who were offered personalized activities at the University of Talca’s clinic center, in the same city.

The control group was constituted of a total of 35 typical older adults of both genders and from the same city, enrolled in other community groups and who participated in leisure and physical conditioning activities or other programs that were not based on reminiscence. It should be noted that the implementation of the intervention for the study group was carried out with different temporalities, safeguarding the same conditions and contexts for all participants.

Table 1 summarizes the sociodemographic and clinical history of both groups.

**Table 1:** Sociodemographic and clinical history of older adult participants

Parameters	Study group (n= 65)	Control group (n=35)	P-value^*^
**Age (years old), Mean ± SD**	73.92 ± 8.01	74.09 ± 7.96	***p> 0.05***
**Education level (years), Mean ± SD**	7.37 ± 2.80	8.23 ± 2.65	***p> 0.05***
**Gender, absolute frequency (%)**	M= 14 (21. 54%)	M= 6 (17. 14%)	--
	F= 51 (78.46%)	F= 29 (82. 86%)	
Perception of socioeconomic level	L= 45 (69. 23%)	L= 25 (71. 43%)	--
	M= 20 (30. 77%)	M= 10 (28. 57%)	
ADL functionality			
**PFAQ, Mean ± SD**	2.71 ± 1.02	2.15 ± 0.86	***p> 0.05***
Global cognitive functioning			
**MMSE, Mean ± SD**	26.72 ± 2.05	27.17 ± 1.72	***p> 0.05***

* Values of statistical significance for the U-Mann Whitney test; (N.S) = p> 0.05, non-significant differences

**Caption:** SD= Standard deviation; MMSE= Mini-Mental State Examination; M= masculine; F= female; L= Low socioeconomic level; M= middle socioeconomic level: ADL= Activities of Daily Living; PFAQ= Chilean version of the Pfeffer Functional Activities Questionnaire

### Ethical considerations

The CCSP-R was applied after the approval granted by the Research Ethics Committee of the Federal University of Santa Maria, Brazil, page 3,000,149. All participants also signed a free and informed consent form.

### Statistical analysis

Two outcome measures were established: perceived subjective well-being and global cognitive efficacy; the former is a measure of self-perception and the latter is the result of a cognitive screening test. All data were analyzed using the Statistical Package for the Social Sciences SPSS, version 25, for Apple.

1. **Subjective well-being**: The score obtained on the Satisfaction with Life Scale – SWLS^(23)^ was considered to establish whether there were differences in the self-perception of subjective well-being after implementing the intervention. The SWLS includes five questions and a Likert scale of seven, so the minimum score is five points, and the maximum score is 45. There is no cut-off point. High values are assumed with high subjective well-being. As with the previously described measure, an intra-group comparison was made with the Wilcoxon statistic and an inter-group comparison with the Mann-Whitney U statistic;
2. **Global Cognitive Efficacy**: The result from the MMSE Chilean version was associated with the PFAQ^(21)^, which has a cut-off point of 21/22 to differentiate those with cognitive impairment. To identify the gains in global cognitive functioning, the MMSE score was compared before and after implementing the reminiscence-based intervention program. In addition, to establish whether the observed gains were attributed to the program, the Mann-Whitney U test compared the differences found between the post-test and the pretest for the study group and the control group.

## RESULTS

### CCSP-R validation process

The judges agreed that both the proposed work sessions and the general structure of the program contributed to the cognitive-communicative stimulation of older adults within the reference of a functional model (W=0.762; p=0.019).

### CCSP-R evidence of efficacy

#### Analysis of the differences between the pretest and post-test in the self-perception of subjective well-being

In the SWLS pretest result, the scores of the study group (n=65) varied between 10 and 35 points, with a mean score of 22.82 points and a standard deviation of 6.14 points. In turn, in the post-test, its score ranged between 17 and 35 points, with a mean of 25.82 points and a standard deviation of 4.38 points. Such results indicate a statistically significant difference (W= -6.33; p= 0.000) according to the Wilcoxon test.

In the SWLS pretest, the control group (n= 35) showed a minimum score of 12 and a maximum of 35 points, with a mean of 23.74 points and a standard deviation of 6.71. In the post-test, the range of scores varied between 12 and 34, with a mean score of 23.74 points and a standard deviation of 6.71. According to the comparison by the Wilcoxon test, such results indicate no statistically significant difference between the scores (W= -0.37; p= 0.708).

Table 2 summarizes the information from the study group and the control group regarding subjective well-being.

**Table 2:** Comparison of the perception of subjective well-being before and after the reminiscence-based intervention between both groups of participants

	**Outcome measure SWLS**	**Pretest**	**Posttest**	**P-value** ^*^
Study group (n= 65)	range	10 – 35	17 – 35	p= 0.000^**^
	mean ± SD	22.82 ± 6.14	25.82 ± 4.38	
Control group (n= 35)	range	12 – 35	12 – 34	*p> 0.05.*
	mean ± SD	23.74 ± 6.71	23.6 ± 4.79	

* Values of significance for the Wilcoxon test; (N.S)= p> 0.05, non-significant differences;

** p< 0.05: significant differences

**Caption:** SD= Standard deviation; SWLS= Satisfaction with Life Scale

#### Analysis of the differences between the pretest and post-test in global cognitive functioning

In the MMSE pretest, the scores of the study group (n= 65) varied between 24 and 30 points, with a mean score of 26.72 points and a standard deviation of 1.68. In turn, in the post-test, its scores ranged between 24 and 30 points, with a mean of 27.20 points and a standard deviation of 1.87. According to the Wilcoxon test, such a result indicates a statistically significant difference (W= -4.43; p= 0.000).

In turn, in the MMSE pretest, the control group (n= 35) showed a minimum score of 25 and a maximum of 30 points, with a mean score of 27.17 points and a standard deviation of 1.72. Likewise, in the post-test, the scores ranged between 24 and 30, with a mean of 27.29 points and a standard deviation of 2.34. According to the comparisons by the Wilcoxon test, such results indicate no statistically significant difference between the two scores (W= -1.21; p= 0.225).

Table 3 summarizes the information from the study group and the control group regarding global cognitive efficacy.

**Table 3:** Comparison of global cognitive processing before and after the reminiscence-based intervention between the groups of participants

	**Outcome measure MMSE**	**Pretest**	**Post-test**	**P-value** ^*^
Study group (n= 65)	range	24 – 30	24 – 30	*p< 0.05* ^**^
	mean ± SD	26.72 ± 2.05	27.20 ± 1.87	
Control group (n= 35)	range	25 – 30	24 – 30	*p> 0.05*
	mean ± SD	27.17 ± 1.72	27.29 ± 2.34	

* Values of significance for the Wilcoxon test; (N.S)= p> 0.05, non-significant differences. ** p< 0.05: significant differences

**Caption:** SD= Standard deviation; MMSE= Mini-Mental State Examination

Finally, the Mann-Whitney U test compared the differences between the MMSE pretest and post-test results for the study and control groups and statistically significant differences were found (U= 587; p= 0.002).

## DISCUSSION

From a very superficial reading, autobiographical events activities are designed and formulated focusing only on their facilitation. Nonetheless, these interventions have different dimensions and levels of complexity that are inherent to their structure and organization, which, combined with the participants’ heterogeneity and the proposed objectives reveal important challenges to sustain methodological rigor^(24)^.

The intrinsic variability^(5)^ is one of the most discussed limitations of these programs; therefore, it is essential to highlight the importance of protocolization and systematicity when applying all the structural elements of the intervention sessions. We should highlight that ethical aspects must always be considered in this type of activity, both for managing and developing reminiscence actions and guiding potential solutions to be implemented in any eventuality^(25)^.

Undoubtedly, the measures of global cognitive efficacy and subjective well-being obtained herein are references of order and basal complexity for screening evaluations and unidimensional constructs. On the one hand, we must advance on larger and more sophisticated assessment strategies, but without ignoring the need to implement systematic records of measures related to self-care, quality of life, and subjective well-being, as applied in clinical populations^(26)^.

Even though no indicators of change in linguistic behaviors were included herein, which is a limitation of this study, quantifiable differences can be detected in the syntactic complexity of the discourses and the number of content units, as well as a greater specificity in the construction of memories. So far, such backgrounds have been established by comparing intersubject discursive units.

Thus, it is worth highlighting that based on the classic models of communicative-discourse competence^(27)^ and the current participation conceptions in the design of programs and intervention^(28,29)^, the relevance of personal narratives can be viewed as highly functional alternatives to promote identity, empowerment, and functionality maintenance of in older adults. Thereby, the scope is enlarged from clinical populations to prevention and promotion activities in neurodiversity, clearly towards recognizing the inalienable right to communicative interaction for older adults^(30)^, as well as at any stage of the life cycle.

## CONCLUSION

The CCSP-R is a viable alternative for functional cognitive-communicative stimulation that requires a solid and well-cared implementation, considering interaction skills as a basic requirement for the beneficiaries, regardless of their educational level. Furthermore, evidence on statistically significant greater performance of program participants supports its usefulness and efficacy for the intervention. Consequently, this program can be used in Primary Health Care, as well as in other similar care contexts.
